# High-Fat-Diet Suppressed Ketone Body Utilization for Lipogenic Pathway in Brown Adipose Tissues

**DOI:** 10.3390/metabo13040519

**Published:** 2023-04-05

**Authors:** Masahiro Yamasaki, Shinya Hasegawa, Shotaro Ozaki, Masahiko Imai, Daisuke Saito, Noriko Takahashi

**Affiliations:** Department of Health Chemistry, Hoshi University, Shinagawa-ku, Tokyo 142-8501, Japan

**Keywords:** obesity, brown adipose tissue, ketone body, lipid metabolism, acetoacetyl-CoA synthetase

## Abstract

Brown adipose tissue (BAT) consumes excess lipids and produces lipid metabolites as ketone bodies. These ketone bodies are then recycled for lipogenesis by the enzyme acetoacetyl-CoA synthetase (AACS). Previously, we found that a high-fat diet (HFD) upregulated AACS expression in white adipose tissue. In this study, we investigated the effects of diet-induced obesity on AACS in BAT. When 4-week-old ddY mice were fed a HFD or high-sucrose diet (HSD) for 12 weeks, a significant decrease in *Aacs,* acetyl-CoA carboxylase-1 (*Acc-1*), and fatty acid synthase (*Fas*) expression was observed in the BAT of the HFD group, whereas expression was not affected in the HSD group. In vitro analysis showed decreased *Aacs* and *Fas* expression in rat primary-cultured brown adipocytes following isoproterenol treatment for 24 h. In addition, the suppression of *Aacs* by siRNA markedly decreased the expression of *Fas* and *Acc-1* but did not affect the expression of uncoupling protein-1 (*UCP-1*) or other factors. These results suggested that HFD may suppress ketone body utilization for lipogenesis in BAT and that AACS gene expression may be important for regulating lipogenesis in BAT. Therefore, the AACS-mediated ketone body utilization pathway may regulate lipogenesis under conditions of excess dietary fat.

## 1. Introduction

Obesity is currently a serious global health concern. Dietary fat intake is considered a major risk factor for obesity and associated metabolic diseases [1]. However, a high-fat diet with low carbohydrates and controlled calorie intake has become popular for weight loss [2]. These diets are known as ‘ketogenic diets’ because they increase blood ketone levels as metabolites. Ketogenic diets have been used to treat epilepsy and other psychiatric disorders [3,4]. Ketone bodies, such as acetoacetate and β-hydroxybutyrate, are the major metabolites produced by the β-oxidation of lipids. When dietary fat intake is excessive, the body maintains energy metabolism homeostasis by increasing lipid consumption. During active lipid consumption by β-oxidation, excess acetyl-CoA is converted to ketone bodies. Indeed, high-fat-diet-induced obesity increases hepatic ketogenesis and decreases hepatic lipogenesis [5]. Differential effects of high-fat and other diets have been reported for probiotics and gut microbiota [6,7] but remain unclear for in vivo metabolites, such as ketone bodies. Therefore, we attempted to distinguish differences in ketone body utilization by comparing dietary obesity caused by low-lipids/high-carbohydrates with that caused by low-carbohydrates/high-lipids, which produces high levels of ketone bodies as a metabolite.

To study this difference in dietary obesity, we focused on intracellular ketone utilization pathways. In ketone body utilization, the mitochondrial enzyme succinyl-CoA: 3-oxoacid CoA-transferase (SCOT, EC 2.8.3.5) is responsible for the generation of ATP from ketone bodies [8]. However, we previously identified acetoacetyl-CoA synthetase (AACS, EC 6.2.1.16) as a lipogenic ligase that directly activates acetoacetate, a ketone body in the cytosol [9,10]. AACS is highly expressed in lipogenic tissues, such as the brain, liver, and adipose tissue [10,11,12]. In the liver, AACS knockdown using the hydrodynamics method decreases serum total cholesterol levels [13]. In white adipose tissue (WAT), AACS expression is induced during adipocyte differentiation [12]. The knockdown of AACS by small interfering RNA (siRNA) significantly reduces lipid droplets in adipocytes [14]. These results suggest that ketone bodies are not only lipid metabolites, but also important nutrients, as evidenced by the presence of AACS in many lipogenic tissues. Furthermore, we previously reported that AACS expression increased in high-fat diet-induced obesity, specifically in WAT [15] and osteoclasts [16], whereas it was not affected in rats fed a high-sucrose diet. The data suggest that the AACS-mediated ketone utilization pathway is a key factor involved in the metabolic differences in diet-induced obesity.

Brown adipose tissue (BAT) is important in diet-induced obesity. Unlike WAT, BAT utilizes fat for thermogenesis [17,18]. Therefore, under cold stress conditions, β-adrenergic stimulation from the central nervous system activates uncoupling proteins (UCPs), which activate the β-oxidation of lipids [19]. Moreover, BAT plays an important role in preventing obesity through the consumption of excess lipids. The removal of BAT in rats leads to an excessive accumulation of body fat and BAT dysfunction exacerbates obesity [20,21]. Functionally active BAT is present in adult humans, and a low proportion and activity of BAT in humans can lead to obesity [19,22]. Therefore, detailed studies on energy metabolism in BAT are important for the prevention and treatment of diet-induced obesity and associated metabolic diseases.

There are various theories on how ketone bodies in the circulating blood are utilized in BAT. Large amounts of acetoacetate are metabolized in BAT and converted to CO_2_ [23]. Moreover, β-hydroxybutyrate may be an important lipogenic substrate for BAT in cold-adapted rats [24]. Furthermore, BAT-specific mitochondrial pyruvate carrier-1 (MPC-1) knockout mice displayed increased β-hydroxybutyrate levels in circulating blood and BAT cytosol [25]. Because the knockout of MPC-1 promotes the depletion of pyruvate in mitochondria, a low-carbohydrate/high-fat diet may cause a similar state in BAT. In diet-induced obesity, oral administration of D-β-hydroxybutyrate-(*R*)-1,3 butanediol monoester (ketone ester) increases BAT UCP-1 levels and improves insulin resistance [26]. The data suggest that ketone bodies could be used as an important nutrient source in BAT. However, no studies have been conducted on ketone body utilization as a lipogenic substrate via AACS rather than other metabolic pathways, such as an energy source. Our recent study showed that AACS might be involved in brown adipogenesis [27]. Furthermore, because the utilization of ketone bodies for lipid synthesis may be important in cold stress-activated BAT [24], we hypothesized that AACS would also be affected by diet-induced activation of BAT. To clarify these possibilities, the effects on ketone bodies and their utilization pathways were investigated in animal models of high-fat and high-sucrose diet-induced obesity.

## 2. Materials and Methods

The methods used in this study followed the Hoshi University Animal Experimentation Guidelines and were approved by the President of Hoshi University after review by the Institutional Animal Care and Use Committee (Permit No. 26-108). The experimental design considered animal ethics and minimized the number of animals used.

### 2.1. High-Fat and High-Sucrose Diet in Mice

To induce nutritional obesity, 27 male and 27 female mice (ddY, 4-week old; Tokyo Laboratory Animals Science Co., Tokyo, Japan) were divided into three groups of each sex. The mice were fed a normal (MF; 47.5% carbohydrates, 23.6% proteins, 5.3% lipids; Oriental Yeast Co., Tokyo, Japan), high-fat (F2HFD2; 7.5% carbohydrates, 24.5% proteins, 60.0% lipids; Oriental Yeast Co.), or high-sucrose diet (F2HScD; 73.6% carbohydrates, 11.8% proteins, 2.6% lipids; Oriental Yeast Co.) for 12 weeks. Detailed dietary composition is shown in Supplementary Table S1. On the afternoon of the last day, the animals were anesthetized, and blood samples were collected from the jugular vein for the measurement of plasma glucose and total ketone bodies. The interscapular BAT was excised for RNA extraction.

### 2.2. Measurement of Plasma Glucose and Total Ketone Bodies

Blood glucose concentration was measured using a glucose assay kit (Glucose CII-Test Wako, Wako Pure Chemical Industries, Osaka, Japan). The plasma levels of total ketone bodies were measured using a ketone body assay kit (Ketone Test Sanwa; Sanwa Kagaku Co., Nagoya, Japan).

### 2.3. Cellular Differentiation Induction of Primary Cultured Brown Adipocytes

The interscapular BAT of Sprague-Dawley rats (4 weeks old, male, Tokyo Laboratory Animals Science Co.) was removed and digested in Hanks’ solution (Worthington Biochemical Co., Lakewood, NJ, USA) containing 3.5% bovine blood albumin (Nacalai Tesque, Kyoto, Japan) and 2 mg/mL of collagenase type I (Worthington Biochemical Co.) for 30 min at 37 °C. Brown progenitor adipocytes were obtained by centrifugation at 1000× *g* for 5 min. Cells were suspended in Dulbecco’s Modified Eagle Medium/Nutrient Mixture F-12 Ham (DMEM/F-12; Invitrogen, Waltham, MA, USA) containing 10% fetal bovine serum (Invitrogen) at a density of 800,000 cells/mL and cultured in a 35 mm dish. When the cells were 70–80% confluent, the medium was supplemented with 1 nM triiodothyronine (T3: Sigma-Aldrich, St. Louis, MO, USA), 25 μM insulin (Invitrogen), 0.125 mM indomethacin (Nacalai Tesque), 5 μM dexamethasone (Nacalai Tesque), and 0.5 mM 3-isobutyl-1-methylxanthine (IBMX; Invitrogen). After 48 h, the medium was replaced with 1 nM T3 and 25 μM insulin and the culture was continued. As a control, non-induced cells were cultured in DMEM/F-12 without induction of differentiation. After 72 h, cells were treated with isoproterenol or siRNA.

### 2.4. Isoproterenol Treatment of Primary Cultured Brown Adipocytes

Differentiated primary brown adipocytes were treated with 0.01, 0.1, 1.0, or 5.0 μM isoproterenol (Sigma-Aldrich) on day 4. Twenty-four hours later, total RNA was extracted from the cells and subjected to real-time PCR, and lipids in the cells were stained with Oil Red O. Cell viability was verified by measuring the number of cells with a hemocytometer (Erma Co., Tokyo, Japan) after staining the cells with trypan blue (Gibco™ Trypan Blue Solution, 0.4%; Thermo Fisher Scientific, Waltham, MA, USA). We confirmed that the number of viable cells after isoproterenol treatment was not significantly different compared to the number of cells cultured normally.

### 2.5. Introduction of siRNA against Acetoacetyl-CoA Synthetase in Primary Cultured Cells 

For the AACS knockdown studies, siRNA transfection was performed based on previously reported methods [28,29]. Differentiation was induced in primary brown adipocytes; AACS siRNA (5′-GUU CAG UGG AAU CGU CUA CTT-3′) or nonsense siRNA (AccuTarget^TM^ Negative Control siRNA (Bioneer, Inc., Alameda, CA, USA)) at a final concentration of 10 pM, mixed with Plus Reagent (Invitrogen). After 15 min, Lipofection Reagent (Invitrogen) was added, and cells were incubated for 3 h at 37 °C. Next, half of the medium containing twice the normal volume of serum and insulin was added. Total RNA was extracted 48 h after transfection, and real-time PCR was performed. AACS protein expression in siRNA-transfected cells was determined using western blotting. The siRNA-transfected cells were lysed in RIPA buffer (0.5% NP-40, 0.1% sodium deoxycholate, 150 mM NaCl, and 50 mM Tris-HCl, pH 7.5). Cell lysates were resolved by SDS-PAGE, transferred onto PVDF membrane (Millipore), and probed with anti-AACS (in-house) and anti-β-actin antibodies (MAB8929; R&D systems, Minneapolis, MN, USA). Cell viability was verified by measuring the number of cells with a hemocytometer (Erma Co.) after staining the cells with trypan blue (Gibco™ Trypan Blue Solution, 0.4%; Thermo Fisher Scientific). We confirmed that the number of viable cells after siRNA treatment was not significantly different compared to the number of cells cultured normally.

### 2.6. RNA Extraction

RNA was extracted from the BAT and primary cells. Prior to RNA extraction, BAT was homogenized using a Polytron homogenizer (Ultra Turrax Homogenizer T8; IKA Inc., Osaka, Japan), and primary cultured brown adipocytes were washed three times with phosphate-buffered saline (PBS; Invitrogen). Total RNA was extracted using ISOGEN (Nippon Gene Co., Tokyo, Japan). Briefly, BAT or primary cells were lysed in 500 μL ISOGEN and 0.2 mL chloroform and incubated for 3 min at room temperature. The samples were centrifuged at 10,000× *g* for 15 min, and the aqueous phase was collected. A total of 0.5 mL isopropanol was added to the aqueous phase, incubated for 10 min at room temperature, and centrifuged at 10,000× *g* for 10 min at 4 °C. The RNA precipitate was rinsed with 1 mL of 70% ethanol and dissolved in dH_2_O. The extracted RNA was used for subsequent experiments. Total RNA (4 μg) from tissues or cells was subjected to real-time PCR to assess the expression of AACS and other enzymatic factors using specific primers (Table S2) and analyzed using a Lumivision imager (AISIN SEIKI Co., Kariya, Japan). The amplified transcripts were quantified using the standard curve method, with β-actin serving as an internal control.

### 2.7. Lipid Staining with Oil Red O

The cells were fixed with 3.5% formaldehyde solution for 10 min, rinsed twice with PBS, and incubated with 1.5% Oil Red O (Sigma-Aldrich) in 60% isopropanol (Sigma-Aldrich) for 1 h. Subsequently, the cells were washed three times with PBS and dried to observe lipid droplets. To quantify the differentiated cells, the stained Oil Red O was redissolved in 100% 2-propanol (Wako Pure Chemical Industries), and the absorbance was measured at 510 nm.

### 2.8. Statistical Analysis

The data were integrated using β-actin as the internal standard. The statistical significance of the data was validated using one-way analysis of variance (ANOVA), followed by Dunnett’s multiple comparison test or Student’s *t*-test. Statistical significance was set at *p* < 0.05.

## 3. Results

### 3.1. Lipid and Ketone Body Utilization in BAT Was Decreased by High-Fat-Diet-Induced Obesity

Previous reports have shown that obesity induced by high-fat and high-sucrose diets has opposite effects on serum parameters [30]. Therefore, we hypothesized that obesity induced by high-fat and high-sucrose diets would have different effects on ketone body utilization. Four-week-old male and female ddY mice were divided into three groups: the high-fat diet (HFD) group was fed a diet in which lipids accounted for 60% of the total calories; the high-sucrose diet (HSD) group was fed a diet composed of 50% sucrose; the control (CD) group was fed a normal diet.

After 12 weeks, body weight increased significantly in the high-calorie (HFD and HSD) groups compared to the CD group (Table 1 and Figure S1). Blood ketone body levels were significantly higher in the male and female HFD groups than in the CD group and were slightly higher in the male HSD group. In contrast, there was little difference in blood glucose levels between the male or female HFD and HSD groups (Table 1).

**Figure 1:**
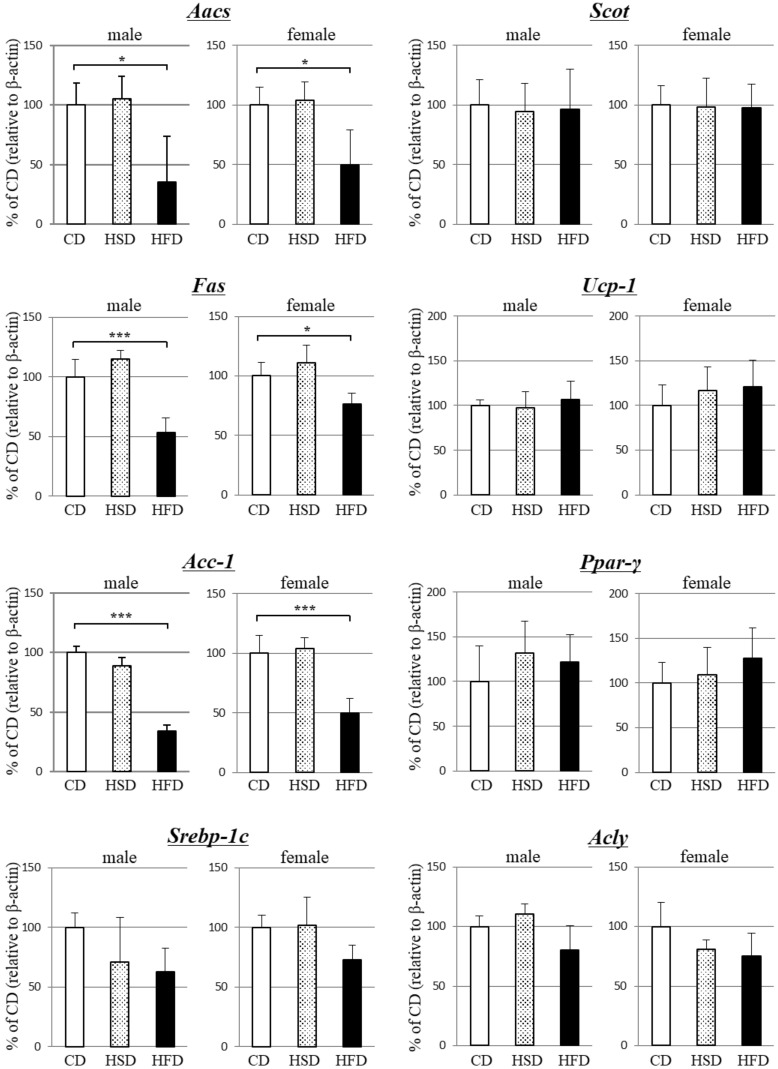
Effect of diet-induced obesity on *gene* expression levels in brown adipose tissue of male and female mice. Total RNA was isolated from interscapular brown adipose tissue and subjected to real-time PCR. The amount of each PCR product was quantified and standardized against β-actin. Mice were fed normal chow (CD), a high-sucrose diet (HSD), or a high-fat diet (HFD) for 12 weeks. The data are expressed as mean ± SD (*n* = 9) and were compared using one-way analysis of variance followed by Dunnett’s multiple comparison test. * p < 0.05, *** p < 0.005 compared with CD group.

Next, we examined the gene expression of various factors in the interscapular BAT of the mice. As shown in Figure 1, *Aacs* expression in BAT was significantly decreased in both male and female HFD mice compared to the CD group, whereas no significant difference was observed in the HSD group. Moreover, a decrease in fatty acid synthase (*Fas*) and acetyl-CoA carboxylase-1 (*Acc-1*) expression was observed only in the HFD group. In contrast, no significant differences were observed in the expression levels of *Scot*, *Ucp-1*, peroxisome proliferator-activated receptor-γ (*Ppar-γ*), sterol regulatory element binding protein-1c (*Srebp-1c*), and ATP citrate lyase (*Acly*) in either the HFD or HSD group. Overall, the data indicated that AACS-mediated lipogenesis might be suppressed by high-fat diet-induced obesity but not by high-sucrose diet-induced obesity.

### 3.2. β-Adrenergic Agonists Suppress Ketone Body Utilization for Lipid Synthesis in BAT

In obese states caused by high-fat diet intake, β-oxidation of fatty acids in brown adipocytes is enhanced by stimulation via β-adrenergic receptors in the central nervous system [31]. Therefore, we investigated whether β-adrenergic stimulation could suppress the expression of *Aacs* using primary cell cultures for this purpose.

Isoproterenol is a non-specific agonist of the β-adrenergic receptor that upregulates lipolysis via activation of UCP-1 [32]. Therefore, we treated primary-cultured brown adipocytes with isoproterenol for 24 h. Treatment with isoproterenol significantly increased ketone body concentrations in the culture supernatant of treated cells compared with that of untreated cells (Table 2).

**Figure 2:**
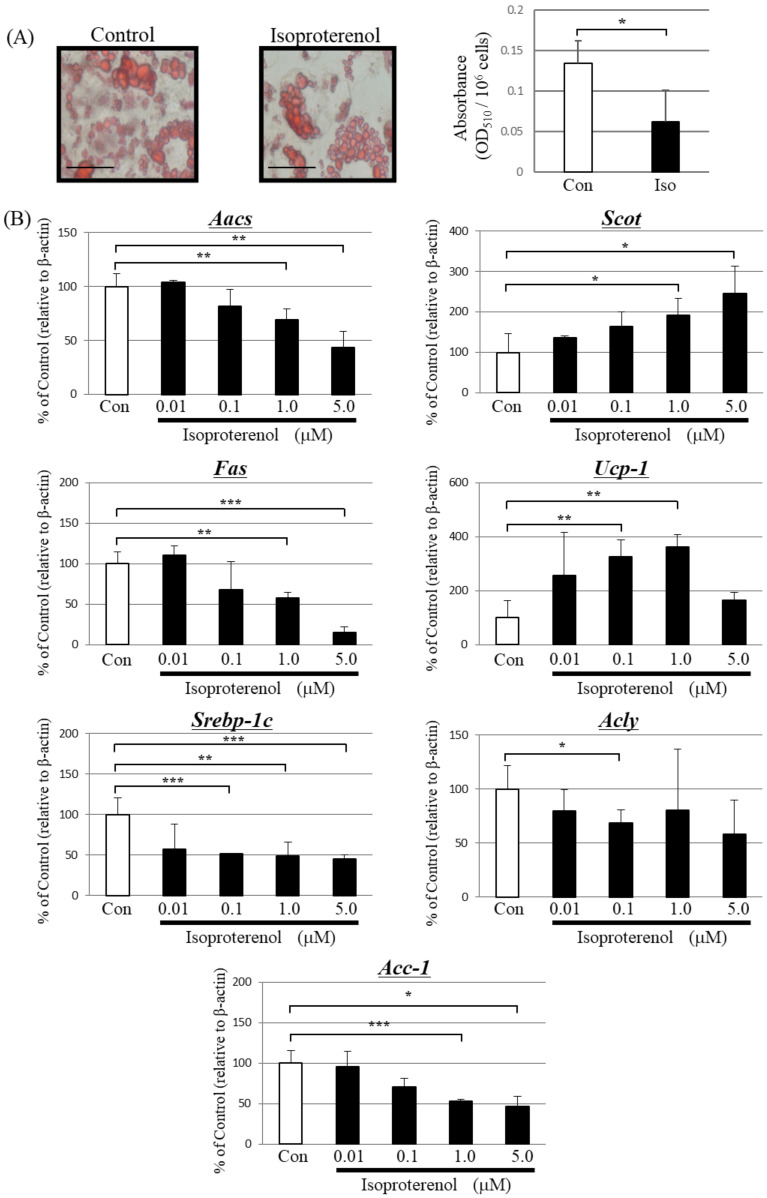
Effect of isoproterenol treatment on differentiated primary brown adipocytes. (**A**) Microscopic view of differentiated primary brown adipocytes with or without 1.0 μM isoproterenol treatment for 24 h (Scale bar, 40 μm). Lipid droplets in cells were stained with the lipophilic dye Oil Red O, and absorbance was measured at 510 nm. Each bar represents the mean ± SD (*n* = 9). Con: vehicle treatment group, Iso: isoproterenol treatment group, * *p* < 0.05. (**B**) PCR analysis for AACS and other enzymes. β-actin was used for standardizing the gene expression levels. The data are expressed as means ± SD (*n* = 9) and were compared using one-way analysis of variance followed by Dunnett’s multiple comparison test. * *p* < 0.05, ** *p* < 0.01, *** *p* < 0.005 compared with control vehicle-treated cells.

Isoproterenol significantly decreased the amount of Oil Red O-stained triglycerides (Figure 2A). Moreover, isoproterenol decreased the expression of *Aacs, Fas,* and *Acc-1* in a concentration-dependent manner compared to the control group (Figure 2B). The expression level of *Srebp-1c* significantly decreased and *Acly* slightly decreased after isoproterenol treatment. In contrast, isoproterenol increased the expression of *Scot* in a concentration-dependent manner and *Ucp-1* expression peaked at 1.0 μM.

### 3.3. Suppression of AACS Expression Significantly Reduced FAS Expression in Primary Cultured Brown Adipocytes

As shown in Figure 2, β-adrenergic stimulation by isoproterenol not only suppressed the expression level of *Aacs* but also affected enzymes in the fatty acid synthesis system. It is unclear whether this effect was due to β-adrenergic stimulation or decreased ketone body utilization via AACS. Therefore, we suppressed *Aacs* expression using siRNA to determine the mechanism of action.

**Figure 3:**
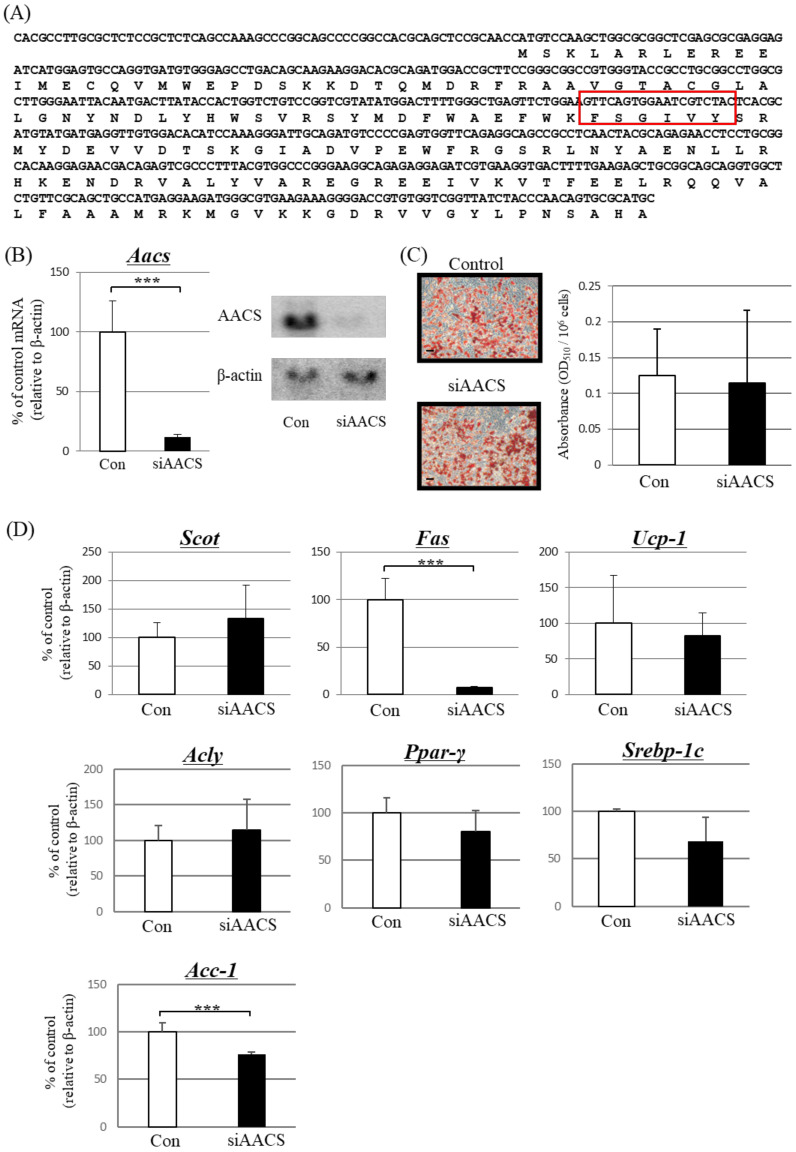
Effect of treatment with siRNA against acetoacetyl-CoA synthetase (AACS) in differentiated primary brown adipocytes. Cells were treated with 10 pM siRNA for AACS (siAACS) or nonsense siRNA (Con) for 48 h. (**A**) Target region of siRNA for the suppression of AACS expression (red square). The top sequence in each line is the nucleotide sequence with amino acids underneath. (**B**) Left panel: mRNA expression of AACS analyzed using real-time PCR. Right panel: Protein expression of cells analyzed using western blotting. (**C**) Microscopic view of differentiated primary brown adipocytes treated with siAACS or Con (Scale bar, 40 μm). Lipid droplets in cells were stained with the lipophilic dye Oil Red O, and absorbance was measured at 510 nm. (**D**) PCR analysis for other investigated factors. β-actin was used for standardizing the gene expression levels. Each bar represents the mean ± SD (*n* = 9). *** *p* < 0.005 compared with control siRNA-treated cells.

Four days after primary-cultured brown adipocytes were induced to differentiate, cells were treated with AACS siRNA (siAACS) targeting the site shown in Figure 3A. After 48 h of siAACS transfection, the mRNA (Figure 3B, left) and protein (Figure 3B, right) expression levels of AACS markedly decreased. In these cells, the expression levels of *Fas* and *Acc-1* notably decreased (Figure 3D). 

Treatment with siAACS did not significantly affect the amount of Oil Red O-stained triglycerides (Figure 3C); however, an increase in the concentration of ketone bodies released from the cultured cells was observed (Table 3). 

In contrast, the expression of *Scot* increased slightly, but the difference was not significant (Figure 3D). No significant changes were observed in the expression of *Ucp-1*, *Acly*, *Ppar-γ*, or *Srebp-1c* (Figure 3D). These results suggested that, even in the absence of β-adrenergic stimulation, the expression levels of *Fas* and *Acc-1* could be affected if AACS was suppressed in brown adipocytes.

## 4. Discussion

In this study, we investigated the differences in obesity between high-fat and high-sucrose diets, focusing on the utilization pathway of ketone bodies, which are lipid metabolites. As previously reported, *Aacs* was specifically upregulated in subcutaneous WAT during high-fat diet-induced obesity [15,33]. In contrast, the present study showed that high-fat diet-induced obesity decreased *Aacs* expression in BAT. The data indicated that *Aacs* expression is differentially regulated in WAT and BAT. Furthermore, these effects were not observed in high-sucrose diet-induced obesity; blood ketone levels were not significantly altered. Although a high-fat diet contained more calories per 100 g than a high-sucrose diet, there was no significant difference in body weight measured between the HSD and HFD groups over our experimental period. Therefore, these changes in BAT were probably not due to differences in the degree of obesity. The results suggested that only ketogenic high-fat diets had significant effects on the expression of *Aacs* and other lipogenic enzymes in BAT. 

Similar to in vivo results in mice fed a high-fat diet, the β-adrenergic stimulation significantly suppressed the expression of *Aacs* and *Fas* in rat primary brown adipocytes in vitro. The data suggest that β-adrenergic stimulation suppresses the utilization of ketone bodies for lipid synthesis. We previously found that high-fat diets resulted in male-specific upregulation of *Aacs* expression in WAT and testosterone-induced *Aacs* expression in 3T3-L1 cells [33]. Although *Aacs* expression in WAT is thought to be regulated by sex hormones, no sex-related differences were observed in *Aacs* expression in BAT. The data suggest the possibility that the expression of *Aacs* in BAT is controlled by β-adrenergic stimulation rather than by sex hormones.

In contrast to *Aacs* and *Fas*, the expression of *Scot* was upregulated upon agonizing the β-adrenergic receptor with isoproterenol treatment. The SCOT-mediated ketone utilization pathway mainly contributes to the oxidative phosphorylation of ketone bodies. Indeed, extracellular ketone bodies are metabolized to CO_2_ in BAT [23]. This result suggests that excess ketone bodies are partially consumed via SCOT in brown adipocytes. In contrast, SCOT was elevated in primary cultured cells, but remained unchanged in animal experiments. This could be due to the possibility that isoproterenol stimulates adrenergic receptors more strongly in vitro than in vivo, or that other factors, such as high concentrations of blood ketone bodies, may be intricately involved in animal experiments.

In our study, suppression of *Aacs* expression also had significant effects on BAT; suppression of *Aacs* expression significantly decreased *Fas* and *Acc-1* expression in primary brown adipocytes. Considering that there were no significant changes in the expression of *Ppar-γ* and *Ucp-1*, the suppression of *Aacs* did not affect the differentiation of brown adipocytes. In addition, *Srebp-1c*, which tightly regulates *Fas* expression [34], was decreased by isoproterenol treatment, but did not significantly decrease in *Aacs*-suppressed cells. Although isoproterenol treatment affected *Fas*, *Acc-1*, *Ucp-1*, and *Srebp-1c*, suppression of *Aacs* only affected gene expression in the fatty acid synthesis pathway. Therefore, the reduced expression of *Fas* in brown adipocytes appears to be dependent, at least partly, on the suppression of ketone body utilization via AACS, rather than being secondary to β-adrenergic receptor stimulation or delayed cell differentiation.

A previous study reported that BAT activation by cold stress increased the expression of *Fas* [35], whereas our data indicated that BAT activation by a high-fat diet decreased the expression of *Fas*. This may be due to differences in ketone body metabolism in BAT. Under a cold-stress state, ketone bodies may be utilized as a lipogenic substrate in BAT [24]. However, our data suggested that a high-fat diet suppressed AACS expression, which may have reduced ketone body utilization for lipid synthesis in BAT. These results may suggest that there are factors other than β-adrenergic stimulation strongly influence the ketone utilization pathway in BAT. In other words, fatty acid synthesis in BAT may be deeply involved not only in the nervous system but also in the AACS-mediated utilization of ketone bodies. The detailed mechanism underlying this regulation is not known; however, several possibilities have been considered. One possibility is that ketone bodies may be involved in post-translational modifications of proteins. Bertolio et al. [36] reported that SREBP-1 signaling is regulated by post-translational modifications. Because acetoacetyl-CoA, which is synthesized from ketone bodies by AACS, is used for lipid synthesis and isoprenoid metabolites, the downregulation of *Fas* in BAT may be involved in post-translational modifications, such as the prenylation of proteins related to the SREBP signaling pathway. Henriksbo et al. [37] reported that statins, inhibitors of the mevalonate pathway, inhibit intracellular signaling by decreasing in isoprenoids required for protein prenylation. 

**Figure 4:**
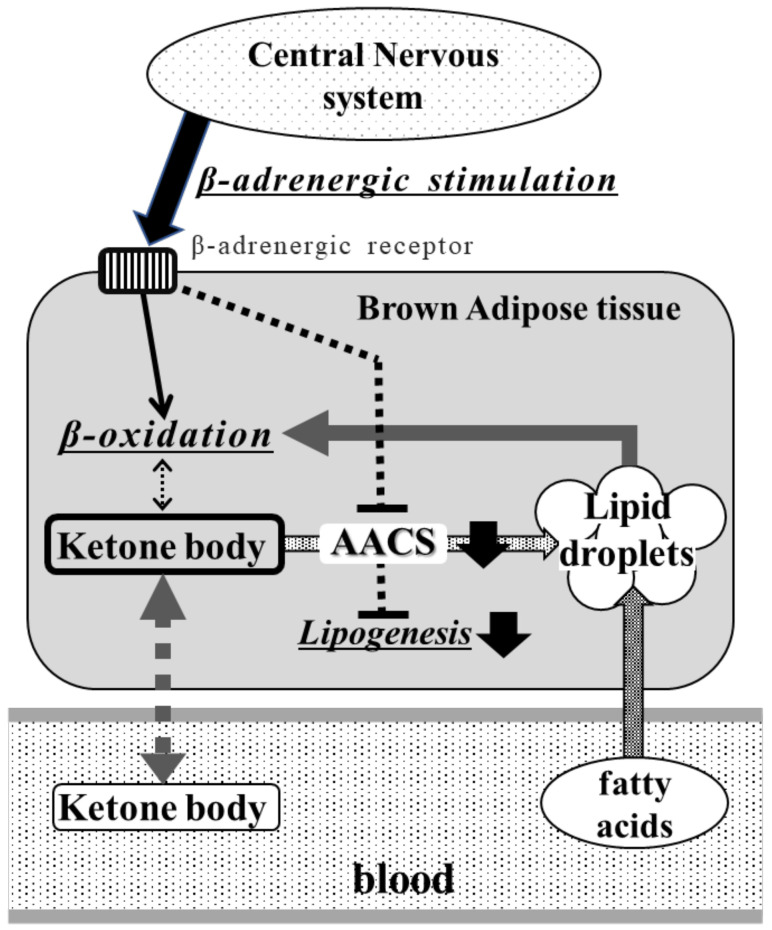
Hypothesized effect of high-fat diets on lipid ketone body metabolism in brown adipose tissue. During high-fat-diet feeding, the β-adrenergic stimulation from the central nervous system enhances the β-oxidation of fatty acids in lipid droplets of brown adipocytes and may suppress AACS expression. This suppression decreases de novo lipogenesis via FAS, reduces the supply of fatty acids to lipid droplets in BAT, and releases ketone bodies into circulating blood. Thus, the decreased lipogenesis might enhance fatty acid uptake from the bloodstream into lipid droplets of BAT.

Lodhi et al. [38] reported that *Fas* expression in adipocytes affects cell fate. Adipocyte-specific FAS-knockout mice promoted the conversion of white adipocytes into brown adipocytes and the activation of BAT, indicating an improvement in high-fat diet-induced obesity and insulin resistance [38]. Therefore, we hypothesized that the suppression of *Aacs* in BAT could lead to the inhibition of new lipid synthesis and the regulation of BAT activation. Moreover, the suppression of lipogenesis in brown adipocytes may contribute to the secondary enhancement of excess dietary lipid uptake in the circulating blood (Figure 4). Huang et al. [39] reported that BAT may play a role as a lipid sink to prevent obesity, and not require thermogenesis for this function. However, this question requires further analyses using BAT-specific knockout mice and other in vivo methods. For these reasons, ketone body metabolism via AACS may play an important role in FAS-mediated lipid metabolism in BAT in high-fat diet-induced obesity.

## 5. Conclusions

High-fat diet-induced ‘ketogenic’ obesity suppressed the AACS-mediated ketone body utilization pathway in BAT through a regulatory mechanism opposite to that in WAT. This suppression was partly mediated by β-adrenergic stimulation. Furthermore, suppression of ketone body utilization may effectively inhibit novel lipid synthesis mediated by FAS in BAT. Similar to rodents, BAT or BAT-like cells (beige adipocytes) are present in adult humans and are considered significant targets against obesity [40]. Therefore, controlling AACS by a high-fat diet with appropriate caloric restriction, such as an appropriate ketogenic diet or pharmaceutical site-specific inhibition of AACS activity, may be useful in the prevention or mitigation of obesity and metabolic syndrome.

## Figures and Tables

**Table 1:** Body weight and blood parameters of diet-induced obese mice. Mice were fed normal chow (CD), a high-sucrose diet (HSD), or a high-fat diet (HFD) for 12 weeks. Data are expressed as mean ± standard deviation (SD; *n* = 9) and were compared with the male or female CD group using one-way analysis of variance followed by Dunnett’s multiple comparison test. ^Ns^: not significant, * *p* < 0.05, *** *p* < 0.001.

	Male Mice			Female Mice		
	CD	HSD	HFD	CD	HSD	HFD
Body weight (g)	44.3 ± 4.1	53.5 ± 8.3 *	59.8 ± 9.7 *	33.1 ± 4.9	38.2 ± 3.7 *	41.4 ± 10.3 *
Blood glucose (mmol/L)	11.2 ± 4.6	14.8 ± 8.4 ^Ns^	15.2 ± 8.2 ^Ns^	11.3 ± 5.1	10.5 ± 3.5 ^Ns^	10.6 ± 5.4 ^Ns^
Total ketone bodies (mmol/L)	0.42 ± 0.19	0.73 ± 0.40 ^Ns^	1.02 ± 0.13 ***	0.27 ± 0.18	0.28 ± 0.13 ^Ns^	1.36 ± 0.43 ***

**Table 2:** Ketone body concentration in the cultured medium of primary brown adipocytes treated with isoproterenol. The data are expressed as mean ± SD (*n* = 9). *** *p* < 0.001.

	Control	Isoproterenol (1.0 μM)
Total ketone bodies (μmol/L)	7.8 ± 10.2	43.6 ± 20.1 ***

**Table 3:** Ketone body concentration in the cultured medium of primary brown adipocytes treated with siRNA against acetoacetyl-CoA synthetase. The data are expressed as mean ± SD (*n* = 9) and were compared using one-way analysis of variance followed by Dunnett’s multiple comparison test. ^ns^: not significant, * *p* < 0.05.

	Control	Nonsense siRNA	AACS siRNA
Total ketone bodies (μmol/L)	7.8 ± 10.2 ^ns^	6.8 ± 12.3 ^ns^	18.6 ± 9.8 *

## Data Availability

Not applicable.

## Supplementary Materials

The following supporting information can be downloaded at: https://www.mdpi.com/article/10.3390/metabo13040519/s1, Table S1: Nutrition in diets. Table S2: Primer sequences for real-time PCR. Figure S1: Body weights of mice with diet-induced obesity.
