# Supplementary material for: High-Fat-Diet Suppressed Ketone Body Utilization for Lipogenic Pathway in Brown Adipose Tissues
Source: Metabolites. 2023 Apr 5;13(4):519. doi: 10.3390/metabo13040519 (PMC10145826; doi:10.3390/metabo13040519)
Supplement: Supplementary file 1 [file metabolites-13-00519-s001.zip › Me01_SUPFig1.pdf]

Figure S1

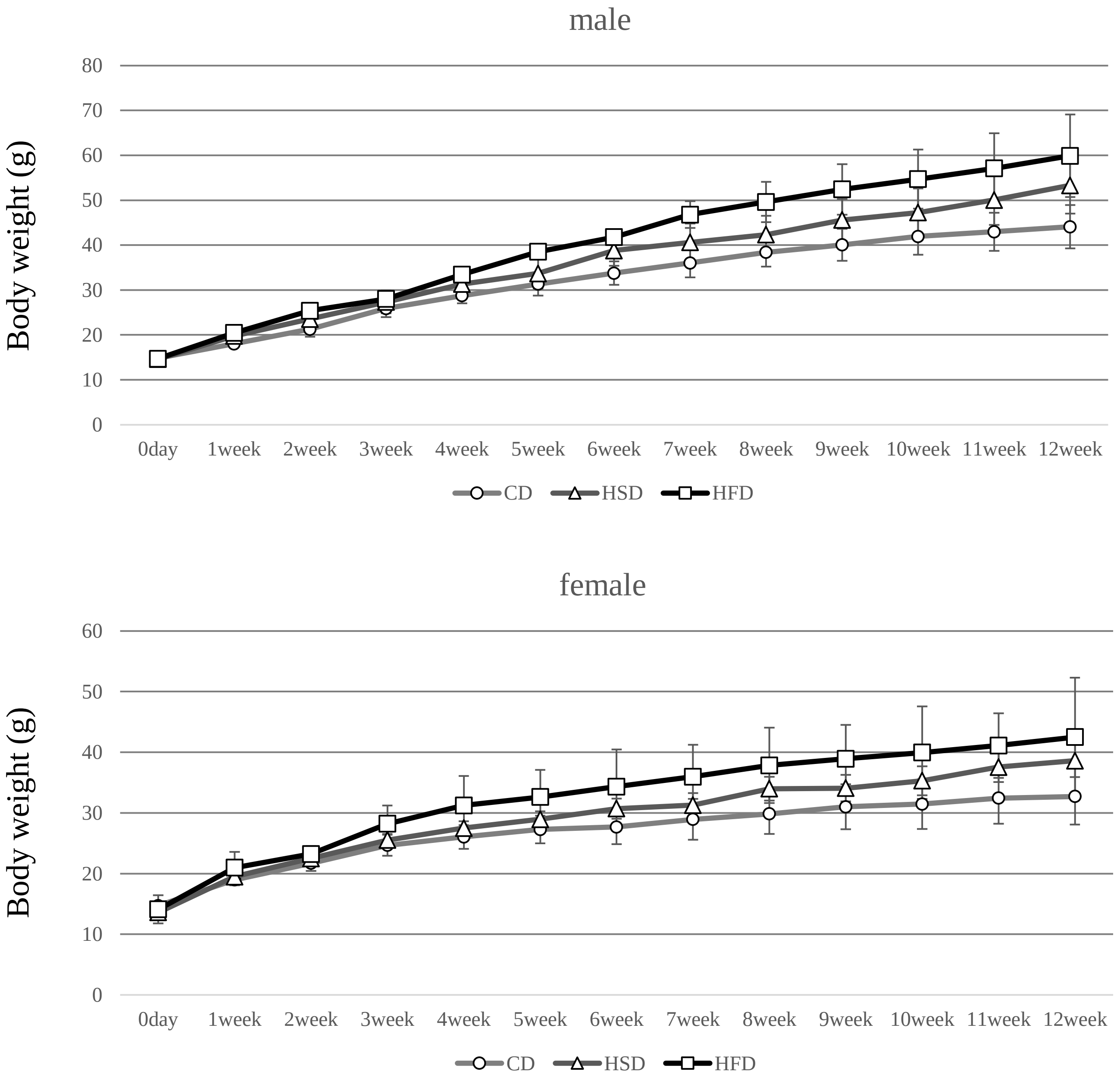

Figure S1. Body weights of mice with diet-induced obesity.

Mice were fed normal chow (CD), high-sucrose (HSD), or high-fat diet (HFD) for 12 weeks. Data are expressed as mean $\pm$ standard deviation (SD; n = 9).
