# Supplementary material for: High-Fat-Diet Suppressed Ketone Body Utilization for Lipogenic Pathway in Brown Adipose Tissues
Source: Metabolites. 2023 Apr 5;13(4):519. doi: 10.3390/metabo13040519 (PMC10145826; doi:10.3390/metabo13040519)
Supplement: Supplementary file 1 [file metabolites-13-00519-s001.zip › Sup_Table_RE01.pdf]

Table S1. Nutrition in diets.

Nutrition (/100g diet)	MF	F2HScD	F2HFD2
Total calories (kcal)	355.7	640.0	370.0
protein (g)	23.2	24.5	12.0
Fat (g)	4.9	60.0	3.0
carbohydrate (g)	54.7	7.5	75.0
Vitamin A (IU)	1638	819	800
Vitamin D_3 (IU)	111	55.5	200
Vitamin E (mg)	8.9	4.5	15.0
Vitamin K_3 (mg)	0.04	0.02	0.02
Vitamin B_1 (mg)	1.9	1.0	1.0
Vitamin B_2 (mg)	1.0	0.5	1.2
Vitamin C (mg)	5.0	2.5	2.5
Vitamin B_6 (mg)	0.9	0.4	1.2
Vitamin B_{12} (μg)	4.6	2.3	5.0
Folate (mg)	0.2	0.1	0.4
Biotin (μg)	30.1	15.1	40.0
Pantothenate (mg)	2.1	1.1	3.0
Niacin (mg)	10.2	5.1	6.0

Table S2. Primer sequences for real-time PCR

	Sense primer	Antisense primer
mouse AACS	ATTCTGGCTCCAGACGATCT	GAATGCAGCCATGTCTATGC
mouse SCOT	CGAAGATGCCGGGCTCTCTC	GATGCTTCAAGTTGAAATCT
mouse UCP-1	ACAGCTGTCTGTCCTACAGA	GTTCATTACGGAACATCTCG
mouse PPAR-γ	GAAACTCTGGGGAGATTCTC	GATCACATGCAGTAGCTGCA
mouse FAS	CCATGGAGGAGGTGGTGATA	CGTCTCGGGGATCTCTCTGC
mouse ACC-1	CAGATAAGGCCTCTGTGCCT	TCACCAGGTTACTGATCTC
mouse SREBP-1c	GGAGCCATGGATTGCACATT	GGCCCGGGAAGTCACTGT
mouse ACLY	GCCCTGGAAGTGGAGAAGAT	CCGTCCACATTCAGGATAAGA
rat AACS	ATGTCCAAGCTGGGCACGGG	GAAGTCCTTTCACGACTCGC
rat SCOT	TTCGAGACACTCGTCGAGAT	AAGATTTATGCCGCTGGGACC
rat UCP-1	GTTCGACAACTTCCGAAGTG	GTTCGACAACTTCCGAAGTG
rat FAS	AACATCACTGAGATTGAGGT	AGTACGCTCAGTGGTAGAAG
rat PPAR-γ	GAGATGGAATTCTGGCCCAC	ACCTTCAATCGGATGGTTC
rat SREBP-1c	GCGCGGACGACGGA	AGTCACTGTCTTGGTTGTTGATGA
rat ACC-1	CTCACTCGAAGCCAGCGTTA	TTGAGGCCAACAGGTTCCAC
rat ACLY	ACCCAGAGGAAGCCTACATTGC	TTCGCCAGTTCGTTGACACC
